# Low-Density Lipoprotein Cholesterol 4: The Notable Risk Factor of Coronary Artery Disease Development

**DOI:** 10.3389/fcvm.2021.619386

**Published:** 2021-04-16

**Authors:** Dongmei Wu, Qiuju Yang, Baohua Su, Jia Hao, Huirong Ma, Weilan Yuan, Junhui Gao, Feifei Ding, Yue Xu, Huifeng Wang, Jiangman Zhao, Bingqiang Li

**Affiliations:** ^1^Department of Cardiovascular Medicine, General Hospital of Tisco, Sixth Hospital of Shanxi Medical University, Shanxi, China; ^2^Department of Cardiovascular Medicine, The First People's Hospital of Pingdingshan, Pingdingshan, China; ^3^Department of Cardiovascular Medicine, Mianxian Hospital, Hanzhong, China; ^4^Shanghai Zhangjiang Institue of Medical Innovation, Shanghai Biotecan Pharmaceuticals Co., Ltd., Shanghai, China

**Keywords:** coronary artery disease, LDL-C subfractions, machine learning, coronary angiography, risk factors

## Abstract

**Background:** Coronary artery disease (CAD) is the leading cause of death worldwide, which has a long asymptomatic period of atherosclerosis. Thus, it is crucial to develop efficient strategies or biomarkers to assess the risk of CAD in asymptomatic individuals.

**Methods:** A total of 356 consecutive CAD patients and 164 non-CAD controls diagnosed using coronary angiography were recruited. Blood lipids, other baseline characteristics, and clinical information were investigated in this study. In addition, low-density lipoprotein cholesterol (LDL-C) subfractions were classified and quantified using the Lipoprint system. Based on these data, we performed comprehensive analyses to investigate the risk factors for CAD development and to predict CAD risk.

**Results:** Triglyceride, LDLC-3, LDLC-4, LDLC-5, LDLC-6, and total small and dense LDL-C were significantly higher in the CAD patients than those in the controls, whereas LDLC-1 and high-density lipoprotein cholesterol (HDL-C) had significantly lower levels in the CAD patients. Logistic regression analysis identified male [odds ratio (OR) = 2.875, *P* < 0.001], older age (OR = 1.018, *P* = 0.025), BMI (OR = 1.157, *P* < 0.001), smoking (OR = 4.554, *P* < 0.001), drinking (OR = 2.128, *P* < 0.016), hypertension (OR = 4.453, *P* < 0.001), and diabetes mellitus (OR = 8.776, *P* < 0.001) as clinical risk factors for CAD development. Among blood lipids, LDLC-3 (OR = 1.565, *P* < 0.001), LDLC-4 (OR = 3.566, *P* < 0.001), and LDLC-5 (OR = 6.866, *P* < 0.001) were identified as risk factors. To predict CAD risk, six machine learning models were constructed. The XGboost model showed the highest AUC score (0.945121), which could distinguish CAD patients from the controls with a high accuracy. LDLC-4 played the most important role in model construction.

**Conclusions:** The established models showed good performance for CAD risk prediction, which can help screen high-risk CAD patients in asymptomatic population, so that further examination and prevention treatment might be taken before any sudden or serious event.

## Introduction

Ischemic heart disease (IHD) is the leading cause of death worldwide according to World Health Organization statistics, and its incidence has been increasing over decade (1). IHD pathophysiological mechanisms (2) are mainly involved in atherosclerosis, coronary microvascular dysfunction, inflammation, and vasospasm. Coronary artery disease (CAD) caused by atherosclerosis is the main cause of IHD. Atherosclerosis is a slowly progressing disease, which might be revealed by typical symptoms such as angina pectoris, or an acute event, including sudden death, without any preceding symptoms (3). Hence, it is crucial to screen population at high risk of CAD, and further medical tests, lifestyle changes, or preventive treatment should be proposed before potentially fatal events occur. So far, coronary angiography is the gold standard for CAD diagnosis, but it is an invasive method and is unpractical for universal screening.

Dyslipidemia is a well-studied risk factor for atherosclerosis (4). Numerous epidemiologic studies and randomized clinical trials have suggested that elevated low-density lipoprotein cholesterol (LDL-C) is a major cause and the target to be controlled to reduce atherosclerotic cardiovascular disease (ASCVD) risk (5–7). However, in fact, a large proportion of atherosclerosis and CAD patients have normal range of blood LDL-C level. LDL-C particles are heterogeneous and can be classified into seven subfractions according to their density and size (8). Increasing evidence indicates that small dense LDL-C (sdLDL-C) is more atherogenic than large buoyant LDL-C (9). SdLDL-C is composed of LDL-C subfraction 3 (LDLC-3) to LDLC-7, and large buoyant LDL-C (lbLDL-C) is composed of LDLC-1 and LDLC-2. A few scattered studies with small sample size have reported LDL-C subfractions' role on CAD. For example, Chaudhary et al. reported that lbLDL-C was negatively correlated with CAD severity (10). In addition, elevated LDLC-4 level was associated with severe CAD in their research, which recruited 179 consecutive patients with suspected CAD, and LDLC-4 was considered as an independent predictive factor for severe CAD based on the result of multivariate analysis (10). Yet there is still lack of substantial studies to elaborate the effects of detailed LDL-C subfractions (LDLC-1 to LDLC-7) on CAD development and its severity.

In this study, we described the blood lipid profile, including LDL-C subfractions, of CAD patients and controls. Then, risk factors of CAD development and severity were analyzed, and predicted models were constructed to assess CAD risk by machine learning method.

## Methods and Materials

### Study Population

A total of 356 newly diagnosed CAD patients and 164 non-CAD controls were recruited (Table 1) from the Department of Cardiovascular Medicine, the General Hospital of Tisco Affilated to Shanxi Medical University, the First People's Hospital of Pingdingshan, and Mianxian Hospital. The inclusion criterion in this study was suspected patients who underwent coronary angiography. And the exclusion criteria were as follows: (1) having a history of severe cardiovascular diseases such as CAD and stroke, (2) having received lipid-lowering therapy, and (3) inability to understand the research aims of this study. When patients were admitted to the hospitals, essential and clinical information were collected, including age, gender, body mass index (BMI), smoking and drinking history, and disease history such as hypertension, diabetes mellitus, and so on. Hypertension is defined as a systolic blood pressure of 140 mm Hg or more or a diastolic blood pressure of 90 mm Hg or more, or taking antihypertensive medication. Diabetes mellitus was evaluated as follows: a previous diagnosis or typical clinical symptoms of diabetes mellitus with random plasma glucose ≥11.1 mmol/L and/or fasting plasma glucose ≥7.0 mmol/L and/or 2-h plasma glucose after 75-g oral glucose tolerance test ≥11.1 mmol/L.

**Table 1:** Clinical characteristics and blood lipids of controls and CAD patients.

**Characters**	**Controls (*n* = 164)**	**Non-obstructive CAD (*n* = 57)**	**Significant CAD (*n* = 299)**	***P*-value**
Age, years	57.41 ± 13.752	56.21 ± 10.20	60.86 ± 11.04	0.004
Median (range)	55 (27–86)	57 (29–76)	60 (33–87)	
BMI (mean ± SD), kg/m^2^	22.98 ± 4.06	25.81 ± 3.45	25.17 ± 3.58	<0.0001
Gender				<0.0001
Male	75 (45.73%)	29 (50.88%)	223 (74.58%)	
Female	89 (54.27%)	28 (49.12%)	76 (25.42%)	
Hypertension				<0.0001
Yes	39 (23.78%)	30 (52.63%)	177 (59.20%)	
Taking antihypertensive medications	25/39	25/30	160/177	
No	125 (76.22%)	27 (47.37%)	122 (40.80%)	
Diabetes mellitus				<0.0001
Yes	5 (3.05%)	7 (12.28%)	70 (23.41%)	
Taking hypoglycemic drugs	1/5	5/7	38/70	
No	159 (96.95%)	50 (87.72%)	229 (76.59%)	
Smoke				<0.0001
Yes	18 (10.98%)	14 (24.56%)	114 (38.13%)	
No	146 (89.02%)	43 (75.44%)	185 (61.87%)	
Drink				0.028
Yes	14 (8.54%)	12 (21.05%)	47 (15.72%)	
No	150 (91.46%)	45 (78.95%)	252 (84.28%)	
TC (>5.17 mmol/L)	23 (14.02%)	12 (10.53%)	50 (16.72%)	0.449
TG (>1.7 mmol/L)	27 (16.46%)	29 (50.88%)	135 (45.15%)	<0.001
HDL-C (<0.86 mmol/L)	27 (16.46%)	15 (26.32%)	82 (27.42%)	0.027
LDL-C (>3.4 mmol/L)	9 (5.49%)	6 (10.53%)	40 (13.38%)	0.031
LDLC-1 (>57 mg/dl)	0 (0%)	0 (0%)	0 (0%)	/
LDLC-2 (>30 mg/ dl)	6 (3.66%)	7 (12.28%)	33 (7.69%)	0.018
LDLC-3 (>6 mg/dl)	6 (3.66%)	48 (84.21%)	222 (74.25%)	<0.001
LDLC-4 (>0 mg/dl)	2 (1.22%)	48 (84.21%)	248 (82.94%)	<0.001
LDLC-5 (>0 mg/dl)	1 (0.61%)	23 (40.35%)	142 (47.49%)	<0.001
LDLC-6 (>0 mg/dl)	0 (0%)	11 (19.30%)	62 (20.74%)	<0.001
LDLC-7 (>0 mg/dl)	0 (0%)	5 (8.77%)	34 (11.37%)	<0.001

*BMI, body weight index; TC, total cholesterol; TG, triglyceride, HDL-C, high-density lipoprotein cholesterol; LDL-C, low-density lipoprotein cholesterol*.

### Definition of CAD Patients and Controls

The CAD patients were diagnosed by coronary angiography, which were divided into two groups including non-obstructive CAD and significant CAD. Significant CAD is defined as coronary artery stenosis ≥50% in at least one main vessel or its major branches. Non-obstructive CAD was defined as visible plaque resulting in <50% luminal stenosis. The Gensini score method was used to evaluate the severity of CAD (11, 12). Gensini score of CAD patients was calculated according to coronary angiography result (12). The controls were diagnosed by coronary angiography without any luminal stenosis or plaque in main vessels and branches.

### Sample Collection and Laboratory Indices Detection

Blood samples were collected after overnight fasting from all participants before taking coronary angiography and concomitant medications. Then, total cholesterol (TC), triglyceride (TG), high-density lipoprotein cholesterol (HDL-C), LDL-C, serum creatinine (SCr), and other routine detection indexes were tested in the Department of Clinical Laboratory. After having been immediately separated, plasma was subjected to 800×*g* centrifugation for 10 min at 4°C. LDL-C subfractions were then classified and quantified by a well-established method, namely, Quantimetrix Lipoprint LDL System (8) (Quantimetrix Corporation, Redondo Beach, CA, USA) according to the manufacturer's instructions. Briefly, the plasma mixed with liquid loading gel was added to the top of precast 3% polyacrylamide gel tubes. After 30 min of photopolymerization at room temperature, samples were electrophoresed for 1 h. Then, densitometry was performed at 610 nm.

### Statistical Analyses

Statistical analyses were performed using SPSS 19.0 (IBM, NY, USA). Differences of categorical variables in distribution between groups were assessed with χ^2^ or the Fisher exact test, as appropriate. Differences of continuous variables among groups were compared by Mann–Whitney *U* or Kruskal–Wallis *H* test. Correlation analysis was conducted by the Pearson correlation method. Graphical plots were generated using GraphPad Prism 6.0 software (La Jolla, CA, USA) and R Project. Logistic regression (LR) analysis was performed to investigate the risk factors of CAD risk by SPSS software. *P* < 0.05 was considered statistically significant. We used Python version 3.7 as the basic language of the whole model and call numpy, panda, sklearn, xgboost, and Matplotlib libraries to process and model the data. After preprocessing the data with numpy and panda, xgboost was used to analyze the importance of features according to the total gain and to select the features with higher importance. StratifiedKFold was used to divide samples into the training set and the test set. The training set was trained by k-nearest neighbors classifier, LR, the support vector machine, the decision tree (DT) model, the multilayer perceptron network, and the XGBoost model. After the training, each model was evaluated in the test set, and then the accuracy, precision, recall, F1 value, and AUC score of each model were calculated, respectively. The receiver operating characteristic (ROC) curve and precision recall curve (PRC) of each model were drawn to present the judgment ability of the model.

## Results

### Clinical Characteristics of Participants

**Figure 1:**
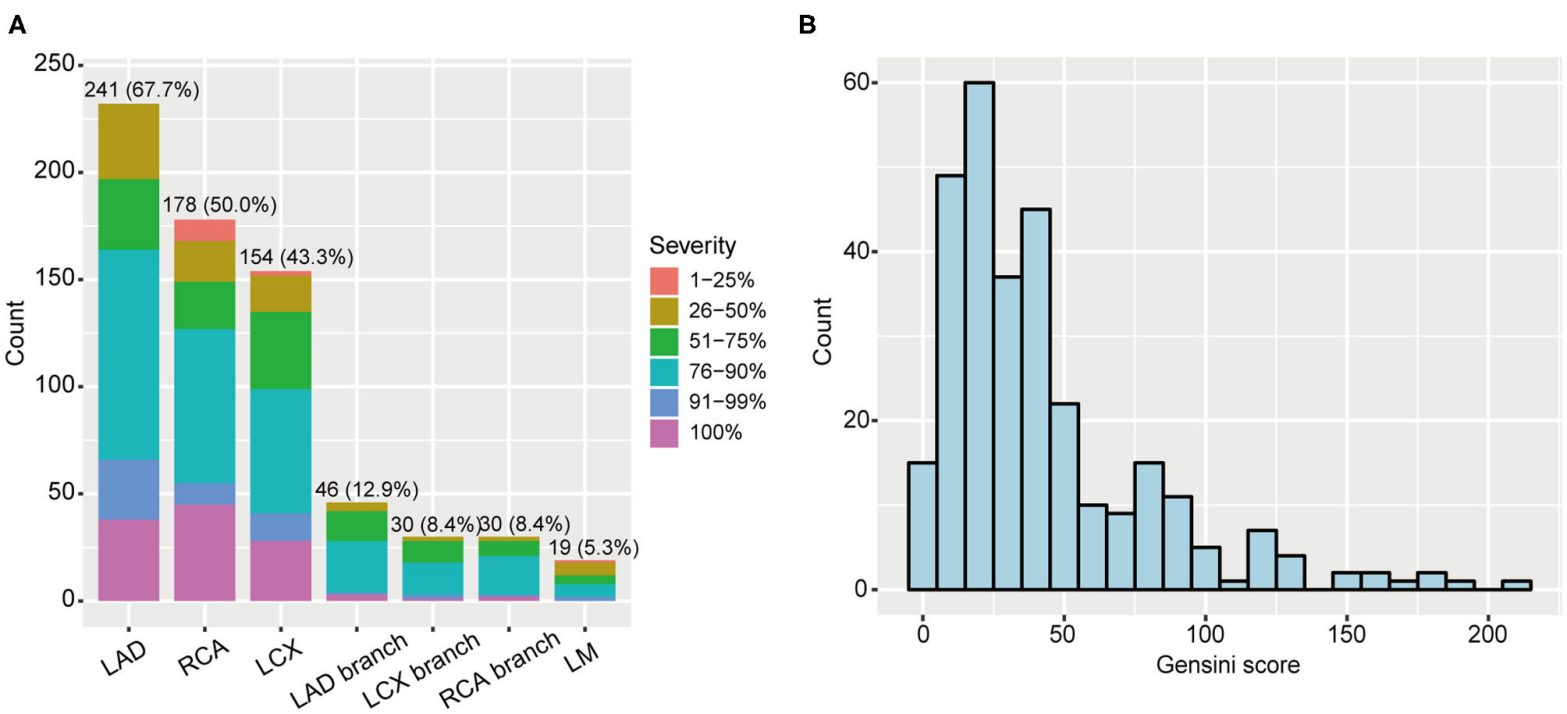
Coronary artery stenosis severity of 356 CAD patients. **(A)** Distribution of stenosis severity and locations. **(B)** Gensini score distribution in CAD patients. CAD, coronary artery disease; LAD, left anterior descending artery; RCA, right coronary artery; LCX, left circumflex artery; LM, left main.

Among 356 CAD patients, 57 and 299 patients were divided into non-obstructive and significant CAD patients, respectively (Table 1). The distribution of coronary artery stenosis severity and location are shown in Figure 1A, from which we can see left anterior descending arteries develop the most frequent stenosis (67.7%) comparing to other vessels. The peak of severity distribution is 76–90%. We also calculated Gensini score of 356 CAD patients, which is shown in Figure 1B.

Table 1 shows the clinical information of 164 controls and 356 first-visit CAD patients. We found that controls and non-obstructive CAD patients had comparative gender ratio, but significant CAD patients had significantly higher ratio of male patients (74.58%). Besides, we also found CAD patients had advantageous distribution in hypertension (*P* < 0.0001), diabetes mellitus (*P* < 0.0001), and smoking (*P* < 0.0001) and drinking history (*P* = 0.028) and had elder age (*P* = 0.004) and higher BMI (*P* < 0.0001) than those in controls.

### Blood Lipid Profile in Controls and CAD Patients

Table 1 shows the abnormal ratio of blood lipids among controls and non-obstructive and significant CAD patients. We found that the abnormal ratios of total LDL-C were only 10.53 and 13.38% in the two groups of CAD patients, which verified that the blood LDL-C levels in a large proportion of CAD patients were in normal ranges. Besides, after comparing with other LDL-C subfractions, LDLC-3 and LDLC-4 have a higher positive rate in non-obstructive (84.21, 84.21%) and significant CAD (74.25, 82.94%) patients.

**Figure 2:**
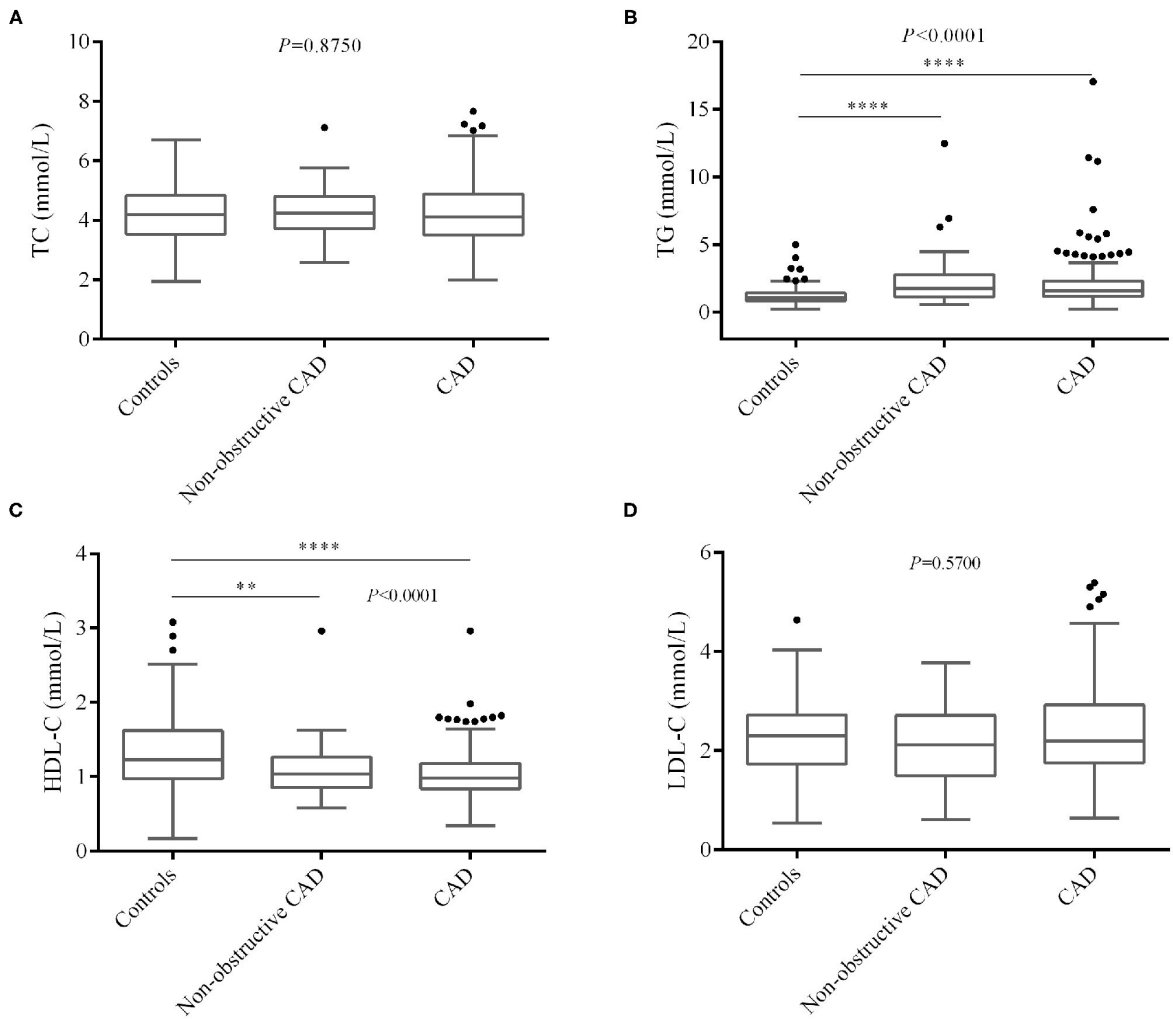
Comparison of blood lipids level among controls and non-obstructive and significant CAD patients. **(A)** TC, **(B)** TG, **(C)** HDL-C, **(D)** LDL-C. CAD, coronary artery disease; TC, total cholesterol; TG, triglyceride, HDL-C, high-density lipoprotein cholesterol; LDL-C, low-density lipoprotein cholesterol. *P < 0.05, **P < 0.01, ***P < 0.001, ****P < 0.0001.

**Figure 3:**
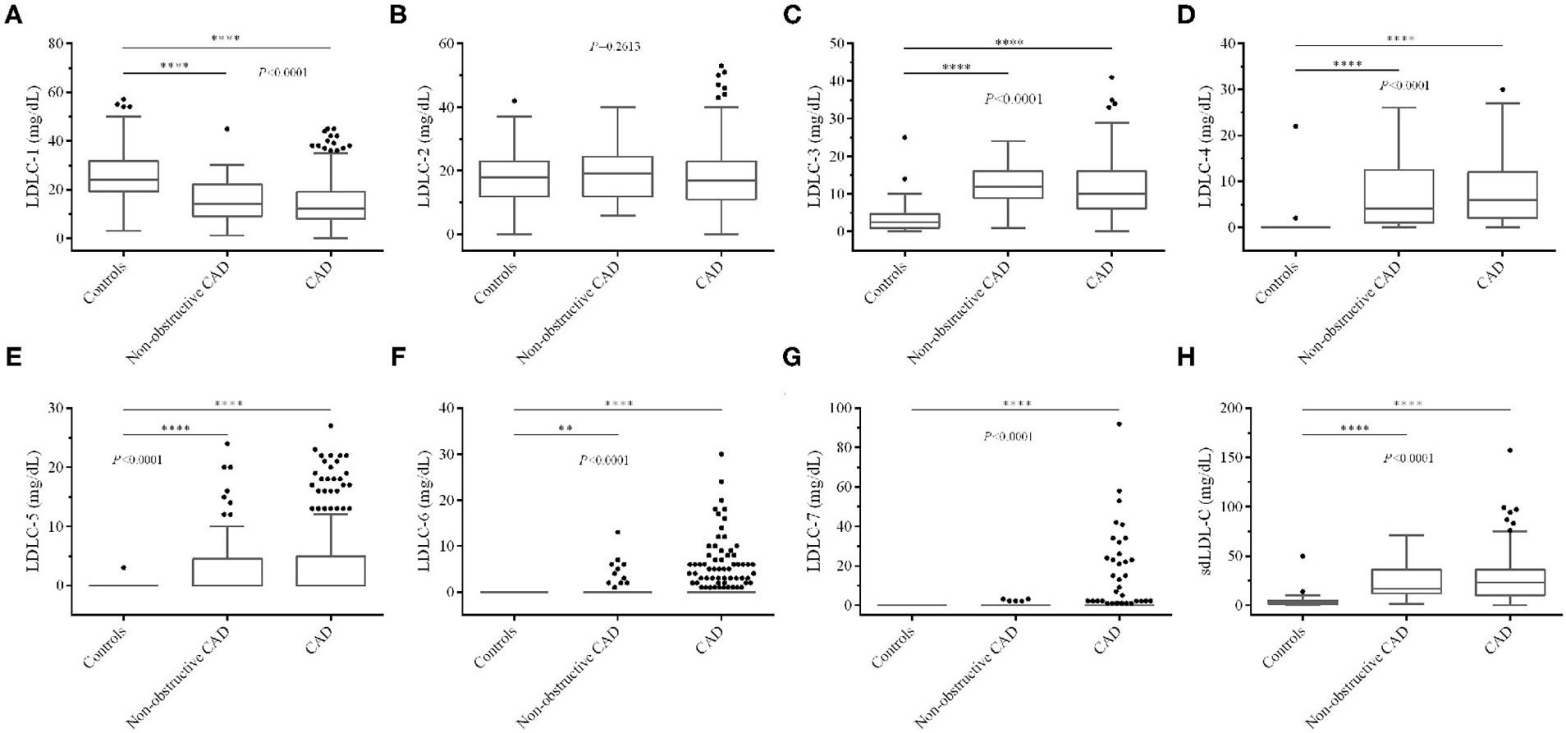
Comparison of LDL-C subfraction levels among controls and non-obstructive and significant CAD patients. **(A)** LDLC-1, **(B)** LDLC-2, **(C)** LDLC-3, **(D)** LDLC-4, **(E)** LDLC-5, **(F)** LDLC-6, **(G)** LDLC-7, **(H)** sdLDL-C. LDL-C, low-density lipoprotein cholesterol; CAD, coronary artery disease. *P < 0.05, **P < 0.01, ***P < 0.001, ****P < 0.0001.

We also compared blood lipid levels among controls and non-obstructive and significant CAD patients, and the results are shown in Figures 2, 3. Surprisingly, there is no significant difference of LDL-C (Figure 2D) between controls and CAD patients. TG is significantly higher (*P* < 0.0001, Figure 2B) and HDL-C (*P* < 0.0001, Figure 2C) is lower in CAD patients than in controls. Figure 3 shows that LDLC-3, LDLC-4, LDLC-5, LDLC-6, and sdLDL-C are significantly higher in CAD patients (*P* < 0.0001), but no significant difference is found between non-obstructive and significant CAD patients. LDLC-1 is higher in controls than those in the two groups of CAD patients, which indicated that LDLC-1 played a protective role in CAD development.

### Correlation Analysis Among Clinical Factors and Blood Lipids

**Figure 4:**
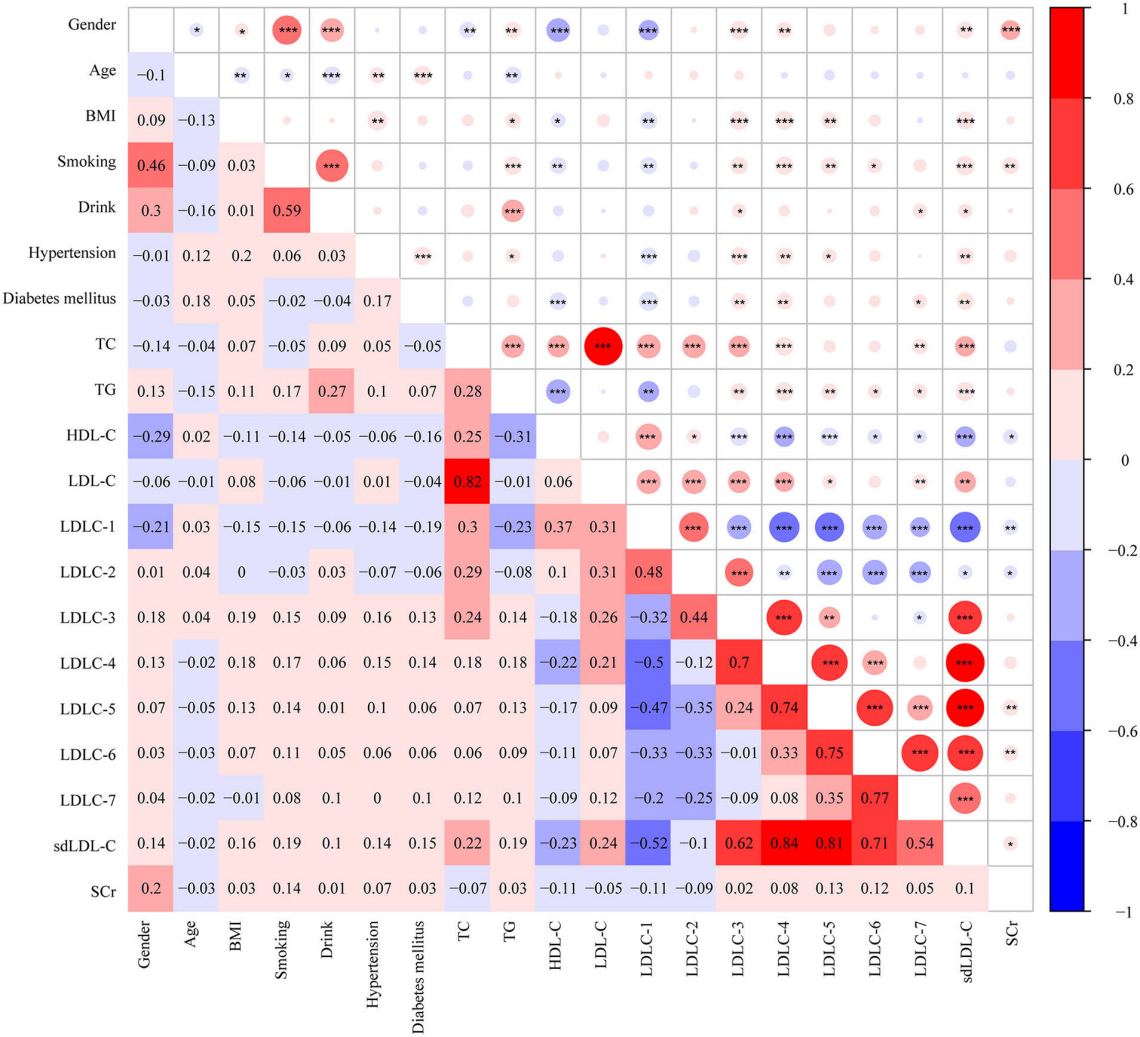
Heatmap showing the correlation among clinical characters and blood markers. The value in grids is Pearson correlation coefficient (Pearson *r*), which is also marked by colors. *P < 0.05, **P <0.01, ***P < 0.001. BMI, body weight index; TC, total cholesterol; TG, triglyceride, HDL-C, high-density lipoprotein cholesterol; LDL-C, low-density lipoprotein cholesterol; sdLDL-C, small and dense LDL-C; SCr, serum creatinine.

Pearson correlation analysis was performed among clinical factors and blood lipids, which is shown in Figure 4. Based on our analyses, LDL-C is strongly positively correlated with TC level (*r* = 0.82, *P* < 0.001). HDL-C is negatively correlated with TG (*r* = −0.31, *P* < 0.001), LDLC-3 (*r* = −0.18, *P* < 0.001), LDLC-4 (*r* = −0.22, *P* < 0.001), and sdLDL-C (*r* = −0.23, *P* < 0.001). A large number of studies have demonstrated that sdLDL-C is negatively correlated with HDL-C concentration (13–15). In our study, we found LDLC-1 is negatively correlated with LDLC-3 (*r* = −0.32, *P* < 0.001), LDLC-4 (*r* = −0.5, *P* < 0.001), LDLC-5 (*r* = −0.47, *P* < 0.001), LDLC-6 (*r* = −0.33, *P* < 0.001), LDLC-7 (−0.2, *P* < 0.001), sdLDL-C (*r* = −0.52, *P* < 0.001), and SCr (*r* = −0.11, *P* < 0.01). We also performed linear correlation analysis between Gensini score of CAD patients and aforementioned blood lipids, and HDL-C was negatively correlated with Gensini score (*r* = −0.178, *P* = 0.001). However, no significant correlation was found in other blood lipids.

### Risk Factors of CAD Development by Logistic Regression

Table 2 shows the results of LR analysis between the subject's factors and CAD risk. For clinical factors, male [odds ratio (OR) = 2.875, *P* < 0.001], older age (OR = 1.018, *P* = 0.025), BMI (OR = 1.157, *P* < 0.001), smoking (OR = 4.554, *P* < 0.001), drink (OR = 2.128, *P* = 0.016), hypertension (OR = 4.453, *P* < 0.001), and diabetes mellitus (OR = 8.776, *P* < 0.001) are identified as CAD risk factors. For blood lipids, LDLC-3 (OR = 1.565, *P* < 0.001), LDLC-4 (OR = 3.566, *P* < 0.001), and LDLC-5 (OR = 6.866, *P* < 0.001) are identified as risk factors for CAD development.

**Table 2:** Logistic regression analysis for independent association between subjects' variables and the risk of CAD.

**Variables**	**Wald**	***P*-value**	**OR**	**95% CI for Exp(B)**	
				**Lower**	**Upper**
Gender	29.238	<0.001	2.875	1.961	4.217
Age, y	5.047	0.025	1.018	1.002	1.034
BMI, kg/m^2^	20.762	<0.001	1.157	1.086	1.231
Smoking	30.803	<0.001	4.554	2.666	7.778
Drink	5.798	0.016	2.128	1.151	3.936
Hypertension	49.368	<0.001	4.453	2.936	6.754
Diabetes mellitus	21.169	<0.001	8.776	3.479	22.139
TC	0.059	0.809	1.024	0.847	1.237
TG	11.112	<0.001	3.128	2.234	4.379
HDL-C	43.983	<0.001	0.155	0.089	0.269
LDL-C	0.443	0.506	1.079	0.863	1.349
LDLC-1	91.835	<0.001	0.891	0.870	0.912
LDLC-2	0.275	0.600	1.006	0.985	1.027
LDLC-3	107.117	<0.001	1.565	1.438	1.704
LDLC-4	51.558	<0.001	3.566	2.520	5.046
LDLC-5	14.450	<0.001	6.866	2.543	18.539

*BMI, body weight index; TC, total cholesterol; TG, triglyceride, HDL-C, high-density lipoprotein cholesterol; LDL-C, low-density lipoprotein cholesterol; OR, odds ratio; CI, confidence interval*.

### Prediction of CAD Risk by Model Established

**Figure 5:**
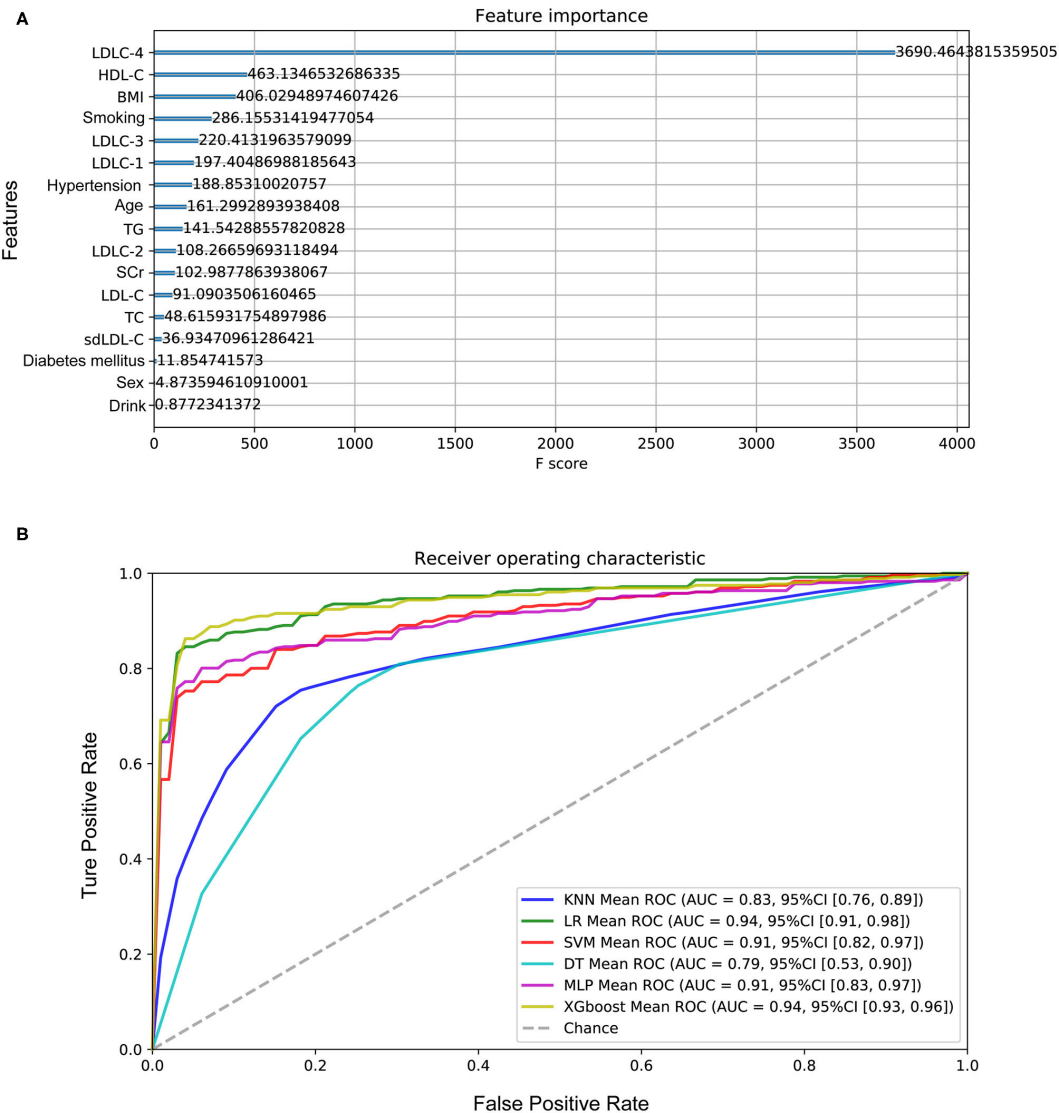
Performance of the models to predict CAD development. **(A)** The importance of features in model construction, including clinical features and laboratory indexes. **(B)** The ROC curves of six models. CAD, coronary artery disease; LDL-C, low-density lipoprotein cholesterol; HDL-C, high-density lipoprotein cholesterol; BMI, body weight index; TG, triglyceride; SCr, serum creatinine; TC, total cholesterol; sdLDL-C, small and dense LDL-C.

One hundred sixty-four controls and 356 CAD patients were grouped, and six models were established to predict CAD risk by machine learning algorithms. The following features were selected according to their importance including LDLC-4, HDL-C, BMI, smoking history, LDLC-3, LDLC-1, hypertension history, age, TG, LDLC-2, SCr, LDL-C, TC, sdLDL-C, diabetes mellitus history, gender, and drinking history, which are shown in Figure 5A. Among all features, LDLC-4 played the most important role in model construction. Table 3 shows the performance of six predictive models. To be specific, LR model is better in accuracy, precision, and F1_score, with the values of 0.880769, 0.920108, and 0.911995, respectively. The DT model gets the highest recall (0.929812). Figure 5B shows the ROC curves of six models, and XGboost has the highest AUC score (0.945121).

**Table 3:** Performance summary of six machine learning models to predict CAD development.

**Models**	**Accuracy**	**Precision**	**Recall**	**F1_score**	**AUC score**
KNN	0.771154	0.833628	0.851056	0.835283	0.827936
LR	0.880769	0.920108	0.907473	0.911995	0.944622
SVM	0.805769	0.877361	0.848318	0.855256	0.907529
DT	0.846154	0.868333	0.929812	0.895352	0.805235
MLP	0.830769	0.889812	0.862285	0.874413	0.907694
XGboost	0.846154	0.878950	0.915806	0.891967	0.945121

*KNN, k-nearest neighbors classifier; LR, logistic regression; SVM, a support vector machine; DT, the decision tree model; MLP, the multilayer perceptron network; AUC, area under curve*.

To further investigate whether CAD severity could be distinguished, we applied similar strategies to establish models between non-obstructive CAD and significant CAD patients. However, the AUC score of the most optimal model in the test set was lower than 0.75, which is not practicable for CAD severity predicted.

## Discussion

In this study, we recruited 356 consecutive CAD patients and a group of controls, which were diagnosed with coronary angiogram. First, we analyzed the correlation between blood lipid profile and CAD risk and found LDLC-3 to LDLC-6, as well as TG, were significantly higher in CAD patients. Total LDL-C showed no significant difference between controls and CAD patients. Several studies reported that LDL-C concentration is not always high in patients with acute coronary syndrome (16–18). LDL-C consists of heterogeneous mixture of particles with different density and size, and sdLDL-C is more atherogenic than lbLDL-C (19, 20). The mechanisms behind this phenomenon may include easier transfer from the vessel lumen into the subintimal space (21), higher affinity to proteoglycans (22), decreased binding ability to LDL-C receptors (23), and increased plasma residence time (24), compared with lbLDL-C.

Belonging to lbLDL-C, LDLC-1 level was relatively lower in CAD patients compared with that in controls, which strengthen the hypothesis that lbLDL-C is a protective factor against CAD development. Chaudhary et al. reported that lbLDL-C was negatively correlated with CAD severity (10). In addition, some other studies also focused on the effects of lbLDL-C on metabolic diseases. For example, Srisawasdi and colleagues' study proposed the ratio of sdLDL-C to lbLDL-C as an excellent biomarker for evaluating lipid metabolic status in patients with metabolic syndrome (25). Chen et al. reported that sdLDL-C/lbLDL-C ratio was associated with glucose metabolic status in pregnancy (26). Interestingly, we also found LDLC-1 was negatively correlated with LDLC-3 to LDLC-7. LDL-C is routinely considered as a major cause of ASCVD risk. However, in the present study, no significant difference was observed in LDL-C levels between controls and CAD groups, and LDL-C was not identified as a significant risk factor for CAD development by LR analysis. A possible explanation could be that the effects of total LDL-C are neutralized by sdLDL-C and lbLDL-C. The possible factors impacting on lipoprotein cholesterol size are genetic background (27, 28) and dietary habits (29–31), but the current evidences were weak. It has great clinical significance on lipid-lowering therapy to explore the mechanisms affecting the size of LDL-C particles.

Silent ischemia occurs in about 20–40% of patients with unstable and stable coronary syndromes (32), and the first clinical manifestation in some cases is sudden events. Thus, we investigated the feasibility of predicting CAD risk by machine learning method. Among six models, XGboost (AUC score = 0.945121) and LR (AUC score = 0.944622) models presented excellent performance for predicting CAD development. Previous researchers (3, 33) had made a lot of efforts to assess cardiovascular risk based on known multiple risk factors. The dominant strategy was calculating the total risk score of a person by summing the risk imparted by each of the major risk factors, for example, Framingham risk score and its improved version (34, 35). By comparison, our study has some advantages. First, we employed a machine learning method, which has superiority on algorithms compared with accumulated risk scores. It could analyze diverse data types (e.g., demographic data, clinical data, laboratory data) and incorporate these features according to their weightiness of information gain into predicted models for disease risk, diagnosis, prognosis, and appropriate treatment (36). In addition, we not only included classical risk factors, but also explored and selected novel marker LDL-C subfractions. Despite that the recent study published by Sánchez-Cabo et al. (37) also used machine learning to cardiovascular risk definition, sdLDL-C or LDL-C subfractions were not considered. Our results showed that LDLC-4 played the most important role on models' establishment, which made a great contribution for better performance of our models than other studies.

Despite the novelties in the present study, there are some limitations worthy of statement. First, numerous patients were excluded, such as patients who received daily lipid-lowering drugs before recruitment, which might limit the generalizability of our finding to wider population. Clinically, many patients with dyslipidemia take cholesterol-reducing medications to prevent atherosclerosis, but some of them still develop CAD. Thus, the efficacy of various lipid-lowering drugs on LDL-C subfractions, especially LDLC-3 and LDLC-4, is worthy of study. Second, in this study, no significant difference was found in blood lipid profile between non-obstructive and significant CAD patients, which is not consistent with the previous study (10). As our study is a cross-sectional study, the blood lipid profile was tested only when participants were recruited. However, CAD development is a long-period progress; both lipid levels and the duration time of dyslipidemia are important factors for atherosclerosis. Thus, this inconsistency might be caused by the lack of continuous monitoring of blood lipid profile changes. Finally, it needs to be mentioned that not only our study but also previous studies came to the conclusion based on a small size of samples. It is necessary to recruit a large cohort of samples to validate these results and perform a longitudinal study with long-time follow-up to give more robust evidence.

## Conclusions

In this study, we proved that LDL-C was insufficient to be a risk factor for CAD development. LDL-C subfractions have shown their importance to be included in the clinical test for screening population with dyslipidemia or high-risk CAD. The established models presented good performance for CAD risk prediction, and LDLC-4 played the most important role in these models, which can help screen high-risk CAD patients in asymptomatic population so that further examination and prevention treatment can be taken before sudden or serious events.

## Data Availability Statement

The original contributions presented in the study are included in the article/Supplementary Material, further inquiries can be directed to the corresponding author/s.

## Ethics Statement

The studies involving human participants were reviewed and approved by the Ethical Committee (the General Hospital of Tisco Affilated to Shanxi Medical University, the First People's Hospital of Pingdingshan and Mianxian Hospital). Written informed consent to participate in this study was provided by the participants' legal guardian/next of kin.

## Author Contributions

DW funded this study. DW, QY, and BL designed project. BS, JH, HM, and HW collected samples and clinical data. WY and JZ performed experiments and analyzed data. DW and JZ wrote the manuscript. BL, HW, JG, FD, and YX revised the manuscript. All authors read and approved the final manuscript.

## Conflict of Interest

WY, JG, FD, YX, and JZ were employed by the company Shanghai Biotecan Pharmaceuticals Co., Ltd. The remaining authors declare that the research was conducted in the absence of any commercial or financial relationships that could be construed as a potential conflict of interest.

## Abbreviations

CAD: coronary artery disease
LDL-C: low-density lipoprotein cholesterol
HDL-C: high-density lipoprotein cholesterol
TC: total cholesterol
TG: triglyceride
SCr: serum creatinine
LR: logistic regression
MLP: the multilayer perceptron network
DT: the decision tree model
SVM: a support vector machine
KNN: k-nearest neighbors classifier
OR: odds ratio
AUC: area under curve.
